# 2025 American Society for Microbiology Awards and Prize Program: honorees from clinical microbiology

**DOI:** 10.1128/jcm.00001-25

**Published:** 2025-02-24

**Authors:** Erik Munson

**Affiliations:** 1 Wisconsin Clinical Laboratory Network Laboratory Technical Advisory Group, Madison, Wisconsin, USA; Boston Children's Hospital, Boston, Massachusetts, USA

## EDITORIAL

Calendar year 2025 will mark the formal recognition of the accomplishments of 20 scientists, educators, and contributors by the American Society for Microbiology (ASM). The American Academy of Microbiology (AAM) is the honorific leadership group within ASM responsible for the management of the ASM Awards and Prize Program, as well as two ASM Lectureships. While a formalized program for awards at ASM has been in existence for approximately 40 years, the first ASM award actually dates back to 1968 when Wayne W. Umbreit received the ASM Carski Award for Undergraduate Education. A listing of all honorees and ASM Lecturers can be found at to https://asm.org/Press-Releases/2024/September/ASM-Selects-Honorees-for-2025-Awards-and-Prize-Pro. In 2025, our discipline is fortunate to sport four honorees within the umbrella of the ASM Awards and Prize Program. Three (including one repeat winner [1] in a different category) will be briefly highlighted in this publication. A fourth, Dr. Benjamin Pinsky of the Stanford University School of Medicine (recipient of the ASM Award for Research or Leadership in Clinical Microbiology, having its roots in the Becton Dickinson and Sonnenwirth awards of the 1970s and 1980s), will be presented in a separate *Journal of Clinical Microbiology* biographical feature.

The ASM Scherago-Rubin Award for Clinical Microbiology was first presented in 1987 and recognizes a non-doctoral level bench microbiologist involved in routine diagnostic work who has distinguished herself by excellent performance. Nominees are further expected to engage with the microbiology community outside of their vocational setting, be it with ASM or other societies of large clinical microbiologist constituency. The award was established by Dr. Sally Jo Rubin in honor of her grandfather, Professor Morris Scherago. The 2025 recipient of the ASM Scherago-Rubin Award for Clinical Microbiology is Carol Lynn Doebler Young, MLS (ASCP), Clinical R&D Project Manager, from the University of Michigan Health in Ann Arbor, Michigan.

Ms. Young earned her baccalaureate degree in Medical Technology at the University of Michigan and completed a clinical internship at the University of Michigan Medical School. In a career that has spanned as long as the publication of this journal, Ms. Young has obviously been the beneficiary of significant advancements in clinical microbiology as both a bench technologist and as a 25-year Laboratory Supervisor, yet her personal statement provides insight into her thirst for personal development, “When I started my career, we had limited guidance documents and as a bench tech I found myself wanting to know more about how our test results could improve our services for our clinicians…I enjoy teaching others and I am a constant learner. I found my niche outside the laboratory at ASM and SCACM (South Central Association for Clinical Microbiology) meetings which gave me ideas to evaluate and implement.”

Ms. Young’s nominator for the award, Dr. James W. Snyder from the University of Louisville Department of Pathology and Laboratory Medicine, cited her career-long role in advancing the science of rapid identification of pathogens from blood culturing. He wrote, “from the development of latex agglutination methods in the late 1980s to the implementation and comparison of the first noninvasive, continuously monitored blood culture systems in the early 1990s (2), Carol went on to advocate for the expansion of the use of blood culture bottles for other sterile body fluids (3), sterility testing, and yeast in the late 1990s. Furthermore, she played a significant leadership role in driving the implementation of various rapid molecular blood culture diagnostic performance improvements in the 2010s.” Ms. Young’s provided *curriculum vitae* lists 55 peer-reviewed abstracts presented at conferences such as ID Week, SCACM, Clinical Virology Symposium, ASM General Meeting, and the Interscience Conference on Antimicrobial Agents and Chemotherapy. Her provided *curriculum vitae* further lists 14 peer-reviewed publications in subjects ranging from chromogenic media (4) to antimicrobial susceptibility testing evaluations (5).

Ms. Young’s interactions with the clinical microbiology community extend to formal presentations given to industry partners, ASM General Meeting sunrise seminar attendees, and SCACM conference-goers. She has served on the Michigan Department of Health and Human Services CRE Surveillance and Prevention Initiatives Committee and coordinated the University of Michigan Health Pathology Laboratory Ambassador Program. Pertaining to SCACM, Ms. Young has served the organization in numerous capacities, including President, Michigan State Director, Corporate Liaison, Workshop Chair, Awards Chair, Nominations Chair, and Exhibits Co-Chair. In 2012, she was recognized as a Significant Contributor to Clinical Microbiology by SCACM.

The ASM Award for Service need not be presented to a clinical microbiologist; yet in recent memory, four experts in our discipline have received the honor (1, 6–8). This year is no different as the 2025 ASM Award for Service is presented to Melissa B. Miller, Ph.D., D(ABMM), Professor of Pathology and Laboratory Medicine at the University of North Carolina School of Medicine in Chapel Hill, North Carolina. Dr. Miller also serves as Director of Clinical Microbiology and Molecular Microbiology Laboratories (McLendon Clinical Laboratories) at UNC Health and was the 2021 recipient of the ASM Award for Research and Leadership in Clinical Microbiology. A Board-certified Medical Technologist by undergraduate training [Jacksonville (FL) University], Dr. Miller completed her doctoral studies at Princeton University by analyzing quorum sensing systems of *Vibrio cholerae* and a Medical and Public Health Microbiology Post-doctoral Fellowship at the University of North Carolina Hospitals thereafter.

The ASM Award for Service recognizes outstanding contributions to the field of microbiology through service. In particular, nominees demonstrate meaningful volunteer service to ASM and its members for multiple years (e.g., editorial board membership, committee service, meeting planning, workshop organization, etc.) without having held elected ASM executive office or currently serving on the ASM Board of Directors. Dr. Robin Patel (9) of the Mayo Clinic in Rochester, Minnesota (and past ASM President) wrote, “I am nominating [Dr. Miller] for her exemplary role as Chair of the ASM Clinical and Public Health Microbiology Committee (CPHMC). The COVID-19 pandemic was a challenging time for everyone…through her leadership role, she advocated for clinical microbiology laboratories and developed guidance for best practices for testing for SARS-CoV-2, which was extraordinarily important given that many laboratories had never liaised with governmental agencies to ensure that the needs of clinical microbiologists and their laboratories were being met. The fast delivery of information and communications was invaluable to the field.” In her personal statement, Dr. Miller recalled, “CPHMC comprises two branches (Clinical and Scientific Practices and Professional Affairs) and 8 subcommittees. Along with ASM staff, I determined this committee’s priorities and long-term strategy. This role required extensive leadership, advocacy, and communication both within ASM and with external partners (10, 11).” Dr. Miller served on CPHMC (formerly known as the Professional Practice Committee) from 2015 to 2023 (Chair from 2018 to 2023). She was co-recipient of the 2021 ASM Special Recognition Award for leadership during COVID-19.

Dr. Miller has served ASM in an array of other capacities. These include the American Board of Medical Microbiology Exam Validation Committee from 2007 to 2010, Scherago-Rubin Award Nominating Committee from 2009 to 2013, Public and Scientific Affairs Board Committee on Laboratory Practices from 2009 to 2017 (Chair from 2015 to 2017), Presidential Transition Advisors Group from 2016 to 2017, Public and Scientific Affairs Committee from 2018 to 2022, Clinical Awards Selection Committee from 2015 to 2019, Committee on Microbial Sciences from 2017 to 2023, two terms on the Clinical Virology Symposium Planning Committee, Next Generation Sequencing Strategic Work Group from 2021 to 2023 (Chair from 2021 to 2022), and the Laboratory Developed Test Working Group (12). Dr. Miller was elected to the AAM Board of Governors in 2024. As an AAM Fellow, she led a symposium on point-of-care testing in 2016 (13). Dr. Miller is currently an editor for *Journal of Clinical Microbiology*, has contributed an editorial pen to two editions of the *Manual of Clinical Microbiology* and one edition of *Molecular Microbiology: Diagnostic Principles and Practice*, and is co-author of the popular ASM Press textbook *Cases in Medical Microbiology and Infectious Diseases* (14). Dr. Miller’s professional service has extended to groups such as American Society for Clinical Pathology, Clinical and Laboratory Standards Institute, College of American Pathologists, Food and Drug Administration, and Pan American Society for Clinical Virology. At the local level, she was bestowed with the North Carolina Governor’s Award for Excellence in Public Service in 2021.

The ASM Award for Early Career Clinical Microbiology Research was first awarded in 2024 (8), recognizing the achievements and research excellence of early-career microbiologists at the doctoral level in either infectious diseases or clinical microbiology. At the time of nomination, the candidate is expected to be within the first 10 years of practice after completion of residency, fellowship, and/or post-doctoral training. The basis for nomination can be a singular seminal, major achievement. or an aggregate of several important findings. Nominees for the award are further expected to promote research in the field of clinical microbiology by way of publications in recognized scientific journals, works presented at (inter)national meetings, and grant awards.

The 2025 recipient of the ASM Award for Early Career Clinical Microbiology Research is Nischay Mishra, Ph.D., Associate Professor of Epidemiology at Columbia University in New York and a virologist in the Columbia University Irving Medical Center’s Center for Infection and Immunity (CII). Dr. Mishra received has baccalaureate degree in Biology from Lucknow University in Lucknow (Uttar Pradesh) India. Advanced degrees in Biotechnology were completed at Chhatrapati Shahu Ji Maharaj University (M.Sc.) in Kanpur (Uttar Pradesh) India and at the National Institute of Virology (Ph.D.) in Pune (Maharashtra) India. Post-doctoral training was completed in 2015 at the Columbia University CII.

Dr. Stephen Morse, Professor of Epidemiology at Columbia University and nominator of Dr. Mishra, described the nominee as having “an exceptional record of accomplishment in developing revolutionary methods for microbial diagnostics. He is not merely content to develop new cutting-edge diagnostic platforms, but is eager to apply them to challenges in diagnostic microbiology. He has been intrepid in tackling difficult diagnostic problems. Using his methods has allowed him to identify novel and previously unidentified disease-causing pathogens, including a disease-associated novel polyomavirus in a transplant recipient (15), and evidence for the role of enterovirus D-68 in acute flaccid paralysis (16).” The latter publication was significant because a peptide microarray detected enterovirus-specific antibodies in 10 patients with acute flaccid paralysis from whom enterovirus-specific nucleic acid was not detected. Such findings assisted in establishing a conceptual foundation for the development of VirCapSeq and BacCapSeq (17), novel paradigms of high-throughput, highly multiplexed platforms to screen for known vertebrate viruses and known human pathogenic bacteria, respectively, which may obviate the need for resource-intensive and time-consuming methods like metagenomics.

Dr. Mishra added in his personal statement, “My methods are extensively deployed in low and middle-income countries across Africa, Asia, and the Americas, providing critical insights that guide outbreak containment and public health.” Completed collaborative grants to the tune of $6.5 million have assisted in this quest. Dr. Mishra currently holds the roles of principal investigator, co-investigator, sub-project principal investigator, or subcontract principal investigator on federal and private funding exceeding $13 million. These efforts have translated into more than 55 peer-reviewed publications, greater than 10 patents, and the United States Food and Drug Administration Emergency Use Authorization approval for two assays. The Triplex CII-SARS-CoV-2 rRT PCR assay for two regions of the nucleocapsid gene and the RNase P housekeeping gene has been utilized in both high-profile settings as well as large school districts for testing and asymptomatic screening. The CII-ArboViroPlex rRT-PCR assay is a one-step test for detection of Zika virus, Chikungunya virus, West Nile virus, and dengue virus (18).

Please join the *Journal of Clinical Microbiology* in congratulating these three microbiologists for their respective contributions.
